# Solid-state fermentation of corn-soybean meal mixed feed with *Bacillus subtilis* and *Enterococcus faecium* for degrading antinutritional factors and enhancing nutritional value

**DOI:** 10.1186/s40104-017-0184-2

**Published:** 2017-06-08

**Authors:** Changyou Shi, Yu Zhang, Zeqing Lu, Yizhen Wang

**Affiliations:** Institute of Feed Science, College of Animal Science, Zhejiang University, Yuhangtang Road 866#, Hangzhou, Zhejiang Province 310058 People’s Republic of China

**Keywords:** Antinutritional factors (ANFs), In vitro digestibility, Mixed feed, Nutritional value, Two-stage fermentation

## Abstract

**Background:**

Corn and soybean meal (SBM) are two of the most common feed ingredients used in pig feeds. However, a variety of antinutritional factors (ANFs) present in corn and SBM can interfere with the bioavailability of nutrients and have negative health effects on the pigs. In the present study, two-stage fermentation using *Bacillus subtilis* followed by *Enterococcus faecium* was carried out to degrade ANFs and improve the nutritional quality of corn and SBM mixed feed. Furthermore, the microbial composition and in vitro nutrient digestibility of inoculated mixed feed were determined and compared those of the uninoculated controls.

**Results:**

During the fermentation process, *B. subtilis* and lactic acid bacteria (LAB) were the main dominant bacteria in the solid-state fermented inoculated feed, and fermentation produced a large amount of lactic acid (170 mmoL/kg), which resulted in a lower pH (5.0 vs. 6.4) than the fermented uninoculated feed. The amounts of soybean antigenic proteins (β-conglycinin and glycinin) in mixed feed were significantly decreased after first-stage fermentation with *B. subtilis*. Inoculated mixed feed following two-stage fermentation contained greater concentratioin of crude protein (CP), ash and total phosphorus (P) compared to uninoculated feed, whereas the concentrations of neutral detergent fiber (NDF), hemicellulose and phytate P in fermendted inoculated feed declined (*P* < 0.05) by 38%, 53%, and 46%, respectively. Notably, the content of trichloroacetic acid soluble protein (TCA-SP), particularly that of small peptides and free amino acids (AA), increased 6.5 fold following two-stage fermentation. There was no difference in the total AA content between fermented inoculated and uninoculated feed. However, aromatic AAs (Phe and Tyr) and Lys in inoculated feed increased, and some polar AAs, including Arg, Asp, and Glu, decreased compared with the uninoculated feed. In vitro dry matter and CP digestibility of inoculated feed improved (*P* < 0.05) compared with the uninoculated feed.

**Conclusions:**

Our results suggest that two-stage fermentation using *B. subtilis* followed by *E. faecium* is an effective approach to improve the quality of corn-soybean meal mixed feed.

**Electronic supplementary material:**

The online version of this article (doi:10.1186/s40104-017-0184-2) contains supplementary material, which is available to authorized users.

## Background

Corn and soybean meal (SBM) are the most common feedstuffs used in pig production in China. However, conventional corn-SBM diets contain a variety of antinutritional factors (ANFs), such as soybean antigenic proteins, phytic acid, oligosaccharides, and other factors that can interfere with the bioavailability of nutrients and have negative health effects in pigs. Soybean antigenic proteins in the diets of weaned pigs provoked a transient hypersensitivity associated with the abnormal morphology of the small intestine [1]. These morphological changes can cause malabsorption syndrome, growth depression, and diarrhea [2, 3]. Phytate in diets may reduce mineral bioavailability and protein digestibility when it is fed to pigs [4]. Furthermore, high levels of soy oligosaccharides, in particular, stachyose and raffinose, can cause intestinal disorder in weaning piglets [5].

Previous research has indicated that fermentation can improve the nutritional quality of animal feed by increasing nutrient bioavailability and reducing ANFs [6]. In China, fermented feed is mainly produced through solid state fermentation (SSF), in which the focus is to decrease the ANFs in single feed ingredients, such as soybean meal [7], cottonseed meal [8], and rapeseed meal [9]. There have been few studies on the use of fermented mixed feed manufactured with SSF. However, the ability of SSF to effectively enhance the nutritional value of mixed feed should be further evaluated. Feeding pigs with fermented liquid feed (FLF) is a useful feeding strategy. Although the growth performance of piglets fed FLF compared with those fed dry feed or non-FLF has been shown to be variable, high lactic acid concentration and low pH in FLF can improve the gastrointestinal health of piglets [10]. In the present study, corn and SBM mixed feed was inoculated with *Bacillus subtilis* in the first stage of fermentation. The aim of the first stage was to decrease ANFs in mixed feed. Subsequently, *Enterococcus faecium* was used in the second stage fermentation to produce lactic acid and reduce the pH of the mixed feed.

## Methods

### Microorganisms and basal substrate

*B. subtilis* ZJ12-1 was isolated from a traditional fermented food (pickled vegetables). *B subtilis* ZJ12-1 was selected with a specific screening plate in which the soybean antigenic protein was the sole nitrogen source extracted from SBM. This strain was identified with Gram's dye and conventional biochemical tests including sugar fermentation, Voges-Proskauer, starch hydrolysis, gelatin liquefaction, salt tolerance etc., then confirmed with 16S rDNA sequencing (Additional file 1: Figures S1, S2, S3 and Table S1). *E. faecium* NCIMB 10415 was obtained from Baolai-leelai Bio-tech Co. Ltd. (Taian, China). *E. faecium*, which is an authorized feed additive for piglets in the EU and China, was isolated from healthy piglet intestines. Dried corn and SBM sieved through 40 mesh sieves were used in SSF.

### Preparation of inoculated mixed feed

**Fig. 1:**
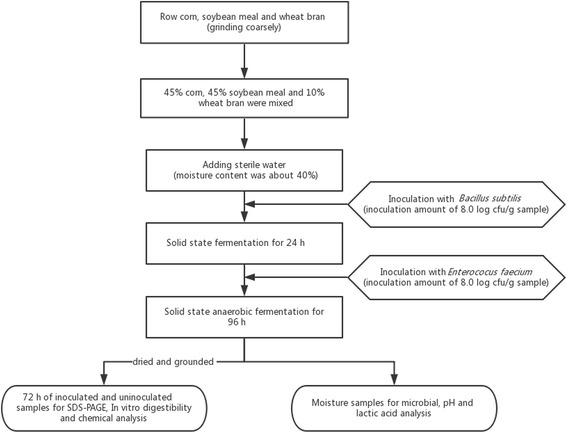
Schematic outline of the manufacturing process for two-stage fermented feed

A schematic outline showing the manufacturing process of the two-stage fermented feed is provided in Fig. 1. Before fermentation, *B. subtilis* was cultured in Luria broth (LB) liquid medium at 37 °C for 12 h. *E. faecium* was cultured in de Man, Rogosa and Sharp (MRS) liquid medium at 37 °C for 16 h. The basal substrate (150 g) included 45% corn, 45% SBM and 10% wheat bran, which was mixed and placed in a 500 mL Erlenmeyer flask covered with cotton plugs and supplemented with sterile water to achieve a 40% moisture content. The wet mixed substrate was inoculated with *B. subtilis* (8.0 log cfu/g) and fermented at 37 °C for 24 h. After the first-stage of fermentation, the fermented mixture was transferred to a plastic bag equipped with a one-way valve (Rou Duoduo Biotechnology Co., Beijing, China), inoculated with *E. faecium* (8.0 log cfu/g), and incubated under anaerobic conditions at 37 °C (the second-stage of fermentation). Uninoculated flasks served as controls. In uninoculated samples, all the experimental procedures were the same as those for inoculated feed, except for the addition of sterile medium (LB and MRS) instead of inoculated bacteria. Inoculated and uninoculated samples (control) were set up in triplicate. After 48 h of anaerobic fermentation, wet samples (approximately 100 g) were collected and treated at 105 °C for 30 min to prevent continuous fermentation. Then, the inoculated and uninoculated samples were dried at 65 °C for 24 h, cooled and ground. Treated samples were subjected to sodium dodecyl sulfate-polyacrylamide gel electrophoresis (SDS-PAGE), in vitro digestibility, and chemical analysis. Moreover, the remaining inoculated feed continued to ferment under anaerobic conditions for 48 h (total 96 h) at 37 °C. Moisture samples were collected at different inoculation times for microbial, pH, and lactic acid analysis.

### Microbial determinations

Inoculated feed at different incubation times (0, 24, 48, 72, 96, and 120 h) were taken, 2 g of samples was diluted 1:9 (w/v) with sterile water. The suspension was homogenized in a stomacher blender (Interscience, St. Nom, France) for 2 min. Ten-fold dilutions were prepared in sterile water and 0.1 mL samples were plated on selective media. Lactic acid bacteria (LAB) were measured on MRS agar following anaerobic incubation at 37 °C for 2 d. The number of *Enterobacteriaceae* was determined on eosin methylene blue agar following aerobic incubation at 37 °C for 1 d. Yeasts and molds were counted on yeast extract peptone dextrose agar with 50 mg/L chloramphenicol (SR0177E, Oxoid LTD, Basingstoke, Hampshire, England) following aerobic incubation at 30 °C for 2 d. Nutrient Broth agar was used to count *Bacillus* spp. by morphological and biochemical identification after aerobic incubation at 37 °C for 1 d. The biochemical tests were the same as strain identification.

### Chemical analysis

Uninoculated and inoculated (24 h with *Bacillus subtilis* and 48 h with *Enterococcus faecium*) feeds were analyzed for dry matter (DM), crude protein (CP), ether extract, neutral detergent fiber (NDF), acid detergent fiber (ADF), ash, calcium (Ca) and total phosphorus (P) using the AOAC International guidelines (2005). The trichloroacetic acid soluble protein (TCA-SP) of the sample was determined using the methods described by Ovissipour et al. [11]. The phytic acid content was measured according to the procedures described by Nair and Duvnjak [12]. Phytate-bound P was calculated as 28.2% of phytate. The amino acid profile was analyzed using an automatic amino acid analyzer (L-8900; Hitachi, Tokyo, Japan). Before analysis, the samples were hydrolyzed with 6 mol/L HCl at 110 °C for 24 h. Methionine and cysteine were analyzed as Met sulfone and cysteic acid after cold performic acid oxidation overnight before hydrolysis. The pH values (at different incubation times) were measured using a HI 99163 pH meter (Hanna instruments, Woonsocket, RI, USA) using 2 g of sample mixed with 18 mL of distilled water. The lactic acid content was determined using a lactic acid enzymology assay kit (Nanjing - Jiancheng Bio Co., Nanjing, China) according to the manufacturer's protocol. The contents of glycinin and β-conglycinin in uninoculated and inoculated feed were analyzed using an indirect competitive enzyme-linked immunosorbent assay (ELISA) kit (Longzhoufangke Bio Co., Beijing, China) according to the manufacturer's protocol.

### Sodium dodecyl sulfate-polyacrylamide gel electrophoresis (SDS-PAGE)

Soluble proteins in fermented uninoculated and inoculated feed were extracted according to the protocol described by Faurobert [13] with some minor modifications. The samples were ground finely to pass through a 60-mesh sieve, and 1.5 mL of Tris–HCl buffer (20 mmol/L, pH 8.3), including 0.1% SDS, 5 mmol/L dithiothreitol and 5 μg/mL protease inhibitor was added to each 0.1 g sample, and then homogenized on ice for 30 min. The homogenized samples were centrifuged at 14,000 × *g* for 10 min at 4 °C (5804R, Eppendorf, Germany), and the supernatants were transferred to Eppendorf tubes. The protein concentration in each sample was determined using the Bio-Rad Protein Assay Kit (Bio-Rad, USA). Soluble protein was fractionated using an SDS-PAGE system as previously described [14]. The electrophoresis system was based on 4 - 12% polyacrylamide gradient separating gels containing 0.1% SDS in Tris-glycine buffer. Approximately 20 μg of extracted protein sample was loaded into each well and separated at 65 mV for 120 min. A Thermo 26616 page ruler pre-stained protein ladder (10–170 kDa) was used as a size marker. After electrophoresis, the gel was stained using Coomassie Brilliant Blue R-250 (Bio-Rad, USA) for 45 min and de-stained with 7% acetic acid.

### In vitro digestibility

An in vitro two-stage enzyme hydrolysis procedure was performed as described by Sakamoto et al. [15], with some modifications. In brief, fermented inoculated feed or uninoculated feed (3 g) was placed in 150 mL Erlenmeyer flasks. Thirty milliliters of 10,000 U/mL pepsin (activity: 3,000 U/mg, Sigma) solution (0.05 mol/L KCl-HCl buffer, pH 2.0) was mixed and incubated at 39 °C at 150 revolutions per min (rpm) for 4 h. The pH was adjusted to 7.0 with 1 mol/L NaOH, and 150 mg trypsin (activity: 250 U/mg, Sigma) was added to each sample, which were then mixed again, and incubated at 39 °C at 150 rpm for 4 h. After the digestion was complete, 5 mL of 20% sulfosalicylic acid was added and the samples were settled for 30 min. The digesta slurry samples were centrifuged at 3,000×g for 15 min, and the supernatants were discarded. The resulting pellets were dried at 105 °C for 4 h and analyzed in subsequent CP and AA assays. In vitro nutrient digestibility (%) = (original nutrient amount – residual nutrient amount) / original nutrient amount × 100%.

### Statistical analysis

The data were analyzed by a one-way analysis of variance using the General Linear Models in SAS software (SAS, 1999). A value of 0.05 was used to indicate of a significant difference. The results are expressed as the means and standard deviations.

## Results

### Microbial composition, pH and lactic acid concentration during SSF

**Fig. 2:**
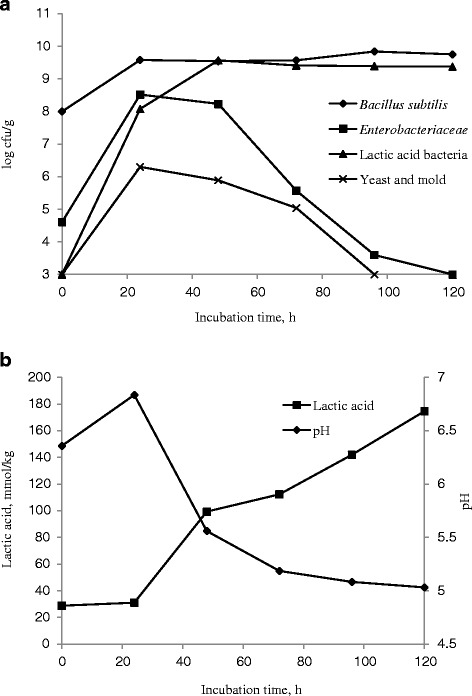
Microbial composition (log cfu/g) **a**, pH, and lactic acid concentration (mmol/kg) (**b**) in inoculated feed during solid-state fermentation, on a DM basis

Figure 2a and b shows the microbial composition, pH and lactic acid dynamics during SSF. When the fermentation was prolonged, *B. bacillus* and LAB were the dominant bacteria present in the solid-state fermented inoculated feed (Fig. 2a). The initial density of *B. subtilis* was 8.0 log cfu/g. After 24 h of incubation, the density increased to 9.6 log cfu/g, and this level was maintained throughout the fermentation experiment. LAB naturally occurring in the mixed feed was low (<3.0 log cfu/g), and the density of LAB at 24 h was 8.1 log cfu/g after inoculation with *E. faecium*; this increased to 9.6 log cfu/g at 48 h in inoculated feed. During the subsequent fermentation period, the number of LAB was similar to that of *B. subtilis*. Notably, there was a proliferation of *Enterobacteriaceae* (potentially pathogens) during the first-stage of fermentation, which reached a maximum level (8.3 log cfu/g) after 24 h of incubation. However, the number of *Enterobacteriaceae* gradually decreased as the fermentation time increased, and the final count was below the level of detection (<3.0 log cfu/g). The two-stage process of *B. subtilis* and *E. faecium* fermentation had a significant effect on both the pH and lactic acid concentrations of the fermented substrate (Fig. 2b). There was a small increase in pH after incubation with *B. subtilis* (6.8 in the inoculated feed with 24 h fermentation vs. 6.4 in the raw mixed feed with 0 h fermentation). After inoculation with *E. faecium*, the pH gradually decreased from 6.8 to 5.0. Almost no change in the lactic acid content was observed during the first-stage of fermentation, and there was a gradual increase in the lactic acid content from 31 to 170 mmol/kg during the second-stage of anaerobic fermentation.

### Biodegradation of soybean antigenic proteins of mixed feed after fermentation

**Fig. 3:**
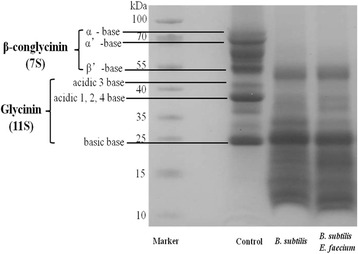
Distribution of peptides in inoculated and uninoculated feed. Marker: protein molecular weight markers (10–100 kDa); control: uninoculated mixed feed (corn 45%, soybean meal 45%, wheat bran 10%) was incubated at 37 °C for 72 h; *B. subtilis*: the mixed feed was inoculated with *B. subtilis* and fermented at 37 °C for 24 h; *B. subtilis* and *E. faecium*: first-stage fermented (*B. subtilis*) mixed feed was inoculated with *E. faecium* and fermented under anaerobic conditions at 37 °C for 48 h

Our results showed that the protein profile corresponded to multiple bands in the range of 23–80 kDa in fermented uninoculated feed (Fig. 3). Subunits of soybean antigenic proteins, including β-conglycinin of α, α´ and β, and acidic and basic glycinin in soybean protein were separated. First-stage fermentation with *B. subtilis* significantly affected the characteristics of proteins in mixed feed. The α and α´ subunits of β-conglycinin and the acidic subunits of glycinin in the mixed feed were almost completely degraded during SSF. In contrast, fermentation increased the number of small peptides (<25 kDa) compared with the fermented uninoculated substrate. However, there was no effect on the protein profile during the second-stage of fermentation with *E. faecium* compared with the first-stage fermented feed. The contents of soybean antigenic protein in fermented uninoculated and inoculated feed are presented in Table 1. Both β-conglycinin and glycinin contents were significantly decreased after fermentation, and degradation of the antigenic protein had already occurred in the first-stage of fermentation.

**Table 1:** Effect of fermentation on the concentration of soybean antigenic protein, as air-dry basis

Item	Glycinin		β-conglycinin	
	Content, mg/g	Degradation^a^, %	Content, mg/g	Degradation, %
Raw mixed feed	63.74	-	31.76	-
Uninoculated feed^b^	61.02	-	32.15	-
*B. subtilis* ^c^	7.97	86.94	6.98	78.28
*B. subtilis* and *E. faecium* ^d^	8.47	86.12	7.12	77.53

^a^Degradation rate = soybean antigenic protein content in uninoculated feed – soybean antigenic protein content in inoculated feed) / soybean antigenic protein content in uninoculated feed × 100%

^b^Uninoculated feed: sterile medium was added to mixed feed (45% corn, 45% soybean meal and 10% wheat bran) instead of inoculated bacteria, other experimental procedures were the same as those of inoculated mixed feed

^c^ *B. subtilis*: mixed feed was inoculated with *B. subtilis* and fermented at 37 °C for 24 h

^d^ *B. subtilis* and *E. faecium*: first-stage fermented (*B. subtilis*) mixed feed was inoculated with *E. faecium* and incubated under anaerobic conditions at 37 °C for 48 h

### Chemical composition

The analyzed nutrient contents of the fermented uninoculated and inoculated feed after 72 h of incubation are presented in Table 2. Compared with uninoculated feed, the inoculated feed contained more CP, ash, and total P, whereas the concentrations of NDF, hemicellulose and phytate P declined (*P* < 0.05) by 39%, 53%, and 46%, respectively. Notably, the content of TCA-SP (<10 kDa) in uninoculated feed was 1.18%, which was increased 6.5-fold in inoculated feed. Inoculating with *B. subtilis* and *E. faecium* affected the AA composition patterns of mixed feed; content of some polar amino acids (Arg, Asp, and Glu) decreased, and aromatic amino acids (Phe and Tyr) and Lys increased. Compared to uninoculated feed, the total AA of inoculated feed increased by 7%; however, the difference was not significant.

**Table 2:** Analyzed nutrient composition of fermented inoculated and uninoculated feed, as air-dry basis^1^

Item	Inoculated feed	Uninoculated feed^2^	AA compositon	Inoculated feed	Uninoculated feed
DM,%	88.06 ± 1.02	89.09 ± 1.67	Indispensable AA, %		
CP,%	27.61 ± 2.73^a^	24.03 ± 1.93^b^	Arg	1.01 ± 0.15^b^	1.17 ± 0.19^a^
TCA-SP,%	8.85 ± 1.19^a^	1.18 ± 0.12^b^	His	0.58 ± 0.08	0.52 ± 0.11
Fat,%	3.37 ± 0.65	3.67 ± 0.73	Ile	0.78 ± 0.13	0.78 ± 0.16
NDF,%	8.33 ± 0.95^b^	13.64 ± 0.99^a^	Leu	1.50 ± 0.18	1.46 ± 0.23
ADF,%	3.58 ± 0.40	3.49 ± 0.76	Lys	1.17 ± 0.08^a^	0.99 ± 0.10^b^
Hemicellulose^3^,%	4.75 ± 0.87^b^	10.15 ± 0.56^a^	Met	0.26 ± 0.05	0.23 ± 0.07
Ash,%	4.71 ± 0.51^a^	3.77 ± 0.38^b^	Phe	1.78 ± 0.26^a^	0.86 ± 0.13^b^
Ca,%	0.18 ± 0.03	0.17 ± 0.02	Thr	0.79 ± 0.12	0.75 ± 0.09
Total P,%	0.55 ± 0.05	0.49 ± 0.07	Val	1.06 ± 0.12	1.04 ± 0.17
Phytate P,%	0.21 ± 0.04^b^	0.39 ± 0.04^a^	Dispensable AA,%		
			Asp	1.68 ± 0.10^b^	1.92 ± 0.17^a^
			Ser	0.79 ± 0.14	0.75 ± 0.16
			Glu	3.23 ± 0.58	3.49 ± 0.44
			Gly	0.85 ± 0.18	0.80 ± 0.13
			Ala	0.98 ± 0.16	0.94 ± 0.10
			Cys	0.48 ± 0.05^a^	0.38 ± 0.06^b^
			Tyr	1.40 ± 0.21^a^	0.67 ± 0.09^b^
			Pro	1.09 ± 0.17	1.17 ± 0.21
			Total AA	19.56 ± 2.33	18.12 ± 2.47

^1^Values are means of three replicates per treatment. Means in a row without common superscript differ significantly (*P* < 0.05)

^2^Uninoculated feed: sterile medium was added to mixed feed (45% corn, 45% soybean meal and 10% wheat bran) instead of inoculated bacteria, other experimental procedures were the same as those of inoculated feed

^3^Hemicellulose = NDF-ADF

### In vitro amino acid digestibility of the fermented samples

The results of in vitro AA digestibility of fermented inoculated feed with two-stage fermentation are presented in Table 3. In vitro CP and DM digestibility of inoculated feed were improved (*P* < 0.05) by 8% and 11%, respectively, compared with uninoculated substrate. In addition, the in vitro digestibility of 11 amino acids, including six essential amino acids (His, Ile, Leu, Met, Phe and Val), improved greatly (*P* < 0.05). Notably, the in vitro digestibility of three amino acids (His, Phe and Cys) increased by more than 10%.

**Table 3:** In vitro CP and AA digestibility (%) of fermented inoculated feed and uninoculated^1^

Item	Inoculated feed	Uninoculated feed^2^
DM,%	70.60 ± 2.87^a^	59.33 ± 2.32^b^
CP,%	86.28 ± 2.23^a^	78.36 ± 2.04^b^
Indispensable AA,%		
Arg	82.50 ± 4.65	82.72 ± 3.87
His	84.91 ± 3.70^a^	74.85 ± 3.46^b^
Ile	80.49 ± 3.42^a^	75.62 ± 2.44^b^
Leu	77.30 ± 3.04^a^	69.71 ± 2.81^b^
Lys	84.59 ± 3.91	81.44 ± 3.60
Met	85.30 ± 3.96^a^	70.31 ± 2.74^b^
Phe	81.99 ± 4.25^a^	65.64 ± 3.63^b^
Thr	78.73 ± 4.12	75.03 ± 3.83
Val	80.74 ± 3.77^a^	74.49 ± 3.48^b^
Mean	81.29 ± 4.09^a^	74.80 ± 3.21^c^
Dispensable AA,%		
Asp	83.14 ± 5.32	78.54 ± 4.97
Ser	77.86 ± 3.13	74.29 ± 3.74
Glu	85.13 ± 2.47^a^	80.25 ± 3.02^b^
Gly	80.78 ± 4.21	76.70 ± 4.08
Ala	84.53 ± 3.38^a^	75.51 ± 3.66^b^
Cys	79.74 ± 3.64^a^	67.90 ± 3.87^b^
Tyr	81.86 ± 3.43^b^	72.28 ± 3.10^c^
Pro	79.31 ± 4.28	75.41 ± 3.94
Mean	82.72 ± 3.11^a^	77.16 ± 3.04^b^
Total AA,%	82.15 ± 3.43^a^	76.07 ± 3.35^c^

^1^Values are means of three replicates per treatment. Means in a row without common superscript differ significantly (*P* < 0.05)

^2^Uninoculated feed: sterile medium was added to mixed feed (45% corn, 45% soybean meal and 10% wheat bran) instead of inoculated bacteria, other experimental procedures were the same as those of inoculated feed

## Discussion

Interest in the fermentation of feed for improving the health of pigs increased dramatically after the European Union banned the use of antibiotics as antimicrobial growth promoters for swine [16, 17]. FLF usually contains >9 log cfu/g of LAB and a high concentration of lactic acid (>150 mmol/L), which can prevent the proliferation of spoilage organisms in the gastrointestinal tracts (GIT) of pigs, such as coliforms and *Salmonella* [18]. Additional advantages of feeding FLF include an increase in nutrient digestibility [19], improved intestinal morphology [20], and a reduction in dust levels in swine barns [21]. Feeding FLF has been shown to improve the performance of piglets and growing-finishing pigs [22], although the results showed high variation. In the present study, changes in the microbial composition with incubation time were determined. During the fermentation process, *B. subtilis* and LAB were the main dominant bacteria in the solid-state fermented feed. The final count of LAB (9.4 log cfu/g) in the present study was similar to that achieved with FLF. The *B. subtilis* count was >9.0 log cfu/g after the first stage of fermentation. However, the source of determined bacteria (exogenous addition or naturally occurring in the feed) is not clear. Notably, a proliferation of *Enterobacteriaceae*, mainly coliforms (potentially pathogens), also occurred at this stage. This result was consistent with that during the initial fermentation of FLF reported by Canibe and Jensen [10]. An increase in feed pH from 6.4 to 6.8 was observed during first-stage fermentation. This increase may be the result of fermentation with *B. subtilis* and other microbes, which introduce some new metabolites or changes in the chemical composition of the substrate. However, additional research is needed to determine the specific reasons for this increase. During the second stage of fermentation, a decrease in pH from 6.8 to 5.0 was most likely the result of increased lactic acid production. Several previously published studies showed that FLF had a pH of 3.8–4.5 [23–25], which was lower than that obtained in the present study. One explanation for the lower pH with FLF may be the difference in the composition of raw materials, since different ingredients have different buffer capacities. The pH may have decreased more rapidly when only the cereals were fermented because cereals have a lower buffering capacity than compound feed [10]. In the present study, the number of *Enterobacteriaceae* gradually decreased as the anaerobic incubation time increased, and the final count was reduced to levels below detection limits (<3.0 log cfu/g). Coliform was reduced mainly due to the low pH and increased lactic acid in fermented inoculated feed. Feeding fermented feed with low pH and high concentration of lactic acid can prevent the proliferation of pathogens (e.g. *Enterobacteriaceae*) along the GIT of piglets [18, 23].

Corn-SBM diet is the most commonly used feed for animal production in China. Corn, as the main energy feed, usually accounts for approximately 60% of the animals’ diet. Soybean meal is the most important plant protein feed for monogastric animals. Soybean antigenic proteins in the diet of weaned pigs, particularly glycinin and β-conglycinin, promote transient hypersensitivity, which may lead to morphological changes in the small intestine, including villi atrophy and crypt hyperplasia [1]. The use of solid-state fermentation to enhance the nutritional characteristics of raw plant materials has been proposed to improve the use of these materials in animal feeds. Several recent studies have shown that soy antigenic proteins could be degraded during fermentation [26, 27]; consequently, immunoreactivity and allergic reactions caused by soy products were reduced in human and animals [28, 29]. In the present study, the first stage of fermentation with *B. subtilis* significantly affected the characteristics of proteins in corn-SBM mixed feed. The α and α’ subunits of β-conglycinin and acidic subunits of glycinin of compound feed were almost completely degraded. This result was consistent with previous reports [14, 27]. ELISA analysis also showed that the contents of β-conglycinin and glycinin in mixed feed were degraded by 78 and 88%, respectively, after the first stage of fermentation. However, no degradation of soybean antigenic protein occurred during the second stage of anaerobic fermentation. This indicated that the decreased amount of antigenic protein in mixed feed may have been due to the hydrolysis of the proteolytic enzyme secreted by *B. subtilis* during the first stage of fermentation. *B. subtilis* secretes many proteolytic enzymes during fermentation, including aminopeptidases, serine endopeptidases, and metalloproteinases [30]. Recently, we showed that the enzyme activity of neutral protease was significantly increased during *B. subtilis* fermentation (unpublished data).

Inoculated mixed feed with two-stage fermentation contained greater concentrations of CP, ash, and total P than uninoculated feed, which was consistent with previous results for fermented compound feed [31], soybean meal [14, 32], rapeseed meal [33, 34] and cottonseed meal [35]. The loss of dry matter (mainly carbohydrates) in the fermented substrate contributing to a relative increase in the concentration of these nutrients was probably the reason for these results. Rozan et al. showed that the loss of dry matter during fermentation may explain the increase in total protein [36]. Because the CP increased after fermentation, the total AA content of inoculated feed would also be increased. However, the composition of amino acids differed between uninoculated and inoculated feed, and some polar amino acids in inoculated feed, such as Asp and Glu were decreased, whereas Tyr, Phe, and Lys increased compared with uninoculated feed. These results were similar to those of a previous study of fermented soybeans [14]. The total protein content in *B. bacillus* is 62.93%. Among them, the most abundant essential AAs are Leu, Lys, Phe, and Val [37], which are more than two times as much as the uninoculated feed. We hypothesized that part of the vegetable protein in mixed feed was used to synthesize microbial protein during SSF. However, the specific mechanism accounting for differences in amino acid composition between fermented inoculated and uninoculated feeds requires further study. Furthermore, inoculated feed exhibited an increase in TCA-SP (8.8%) compared to uninoculated feed (1.2%). An increase in TCA-SP was probably due to the hydrolysis of macromolecular proteins (especially antigenic proteins). TCA-SP was assumed to consist of small molecular peptides (2–20 amino acid residues) and free AAs [38]. Di- and tripeptides in TCA-SP can be directly absorbed in the animal gut system, and transport of AA in the form of small peptides was faster than their constituent AAs in the free form [39]. Furthermore, a decrease in NDF, hemicellulose, and phytic acid in FMF was observed after fermentation. This is might be due to the production of relevant enzymes by micro-organism, such as non-starch polysaccharide (NSP)-degrading enzymes and phytase, which caused the breakdown of these antinutritional substrates. Fermentation of rapeseed meal with *Aspergillus niger* was studied by Shi et al. [33], who showed that the levels of NDF, glucosinolates, and phytic acid in rapeseed meal declined by 14.45%, 43.72%, and 86.08%, respectively, after SSF. Pig feeding showed that fermented rapeseed meal had a greater P and energy digestibility than rapeseed meal [34]. Therefore, lower NDF and phytic acid indicate that inoculated mixed feed with two-stage fermentation may have higher nutrient digestibility compared with non-fermented mixed feed. The results of in vitro digestibility showed that two-stage fermentation with *B. subtilis* and *E. faecium* may improve the nutritional value of corn-SBM mixed feed.

## Conclusions

Two-stage fermentation with *B. subtilis* followed by *E. faecium* effectively reduced ANFs (soy antigenic protein, NDF, and phytic acid) in corn-SBM mixed feed and increased the TCA-SP and CP content. Furthermore, the high lactic acid concentration and low pH in fermented inoculated feed inhibited the proliferation of *Enterobacteriaceae*. The results of in vitro digestion indicated that inoculated feed subjected to two-stage fermentation had higher DM and CP digestibility than fermented uninoculated feed. Therefore, two-stage fermented feed may be used as a novel feed ingredient in animal diets, especially for piglets. Our results suggest that the two-stage SSF method provides an effective approach for improving the quality of corn-soybean mixed feed.

## Additional file

Additional file 1: Strain identification information. (DOCX 1027 kb)

## Abbreviations

AA: Amino acid
ANFs: Antinutritional factors
Ca: Calcium
CP: Crude protein
FLF: Fermented liquid feed
LAB: Lactic acid bacteria
NDF: Neutral detergent fiber
NSP: Non-starch polysaccharide
P: Phosphorus
SBM: Soybean meal
SDS-PAGE: Sodium dodecyl sulfate – polyacrylamide gel electrophoresis
SSF: Solid state fermentation
TCA-SP: Trichloroacetic acid soluble protein
