# Supplementary material for: Solid-state fermentation of corn-soybean meal mixed feed with Bacillus subtilis and Enterococcus faecium for degrading antinutritional factors and enhancing nutritional value
Source: J Anim Sci Biotechnol. 2017 Jun 8;8:50. doi: 10.1186/s40104-017-0184-2 (PMC5465572; doi:10.1186/s40104-017-0184-2)
Supplement: Additional file 1: — Strain identification information. (DOCX 1027 kb) [file 40104_2017_184_MOESM1_ESM.docx]

Fig S1: Morphological characteristic of *Bacillus* spp


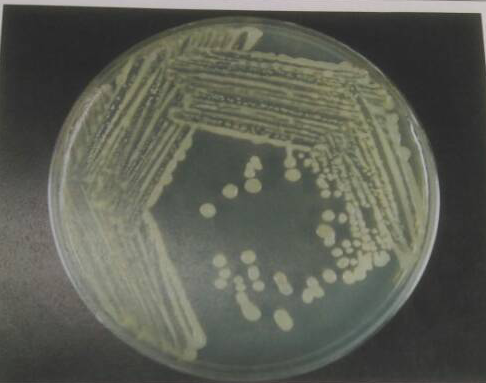


Colony morphology


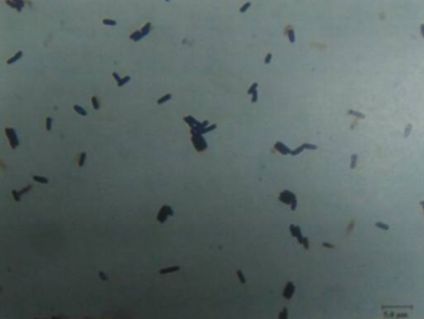


Micromorphology

Table S2: biochemical tests of *Bacillus* spp

| Item | [Identification result](http://dict.cnki.net/dict_result.aspx?searchword=%e9%89%b4%e5%ae%9a%e7%bb%93%e6%9e%9c&tjType=sentence&style=&t=identification+result) |
| --- | --- |
| Contact enzyme | + |
| Anaerobic growth | - |
| VP test | + |
| Sugar fermentation (Glucose, xylose, [arabinose](https://www.baidu.com/link?url=arh-Su-CdT6arZ9OgXtUjTBknZeMsieHpeVSKjyhxL6qYsD3wDNCkLIBoT6c7NmZ-RMOUDsmNKr_1sB0O953vmMQn3C1m5NMRTsrnukFJ67&wd=&eqid=f88c2bb600007beb0000000458d8c581), mannitol) | + |
| Gas production by glucose | - |
| Nitrate reduction | + |
| 6.5% Nacl growth | + |
| Starch hydrolysis | + |
| Gelatin liquefaction | + |

“+”: positive; “-”: negative

Fig S2: 16S rDNA sequencing information of this bacterium


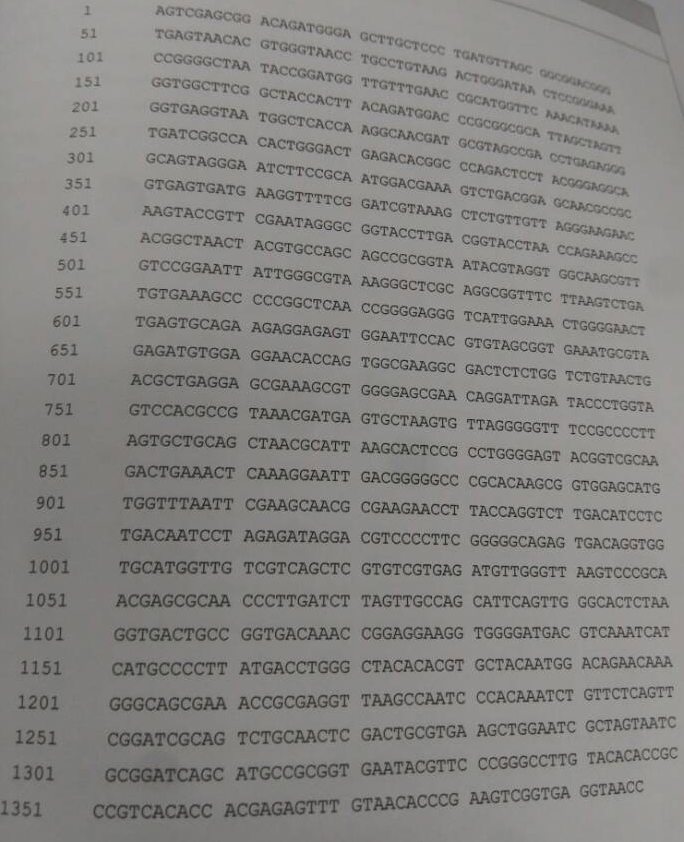


Fig S3: phylogenetic tree analysis of this bacterium


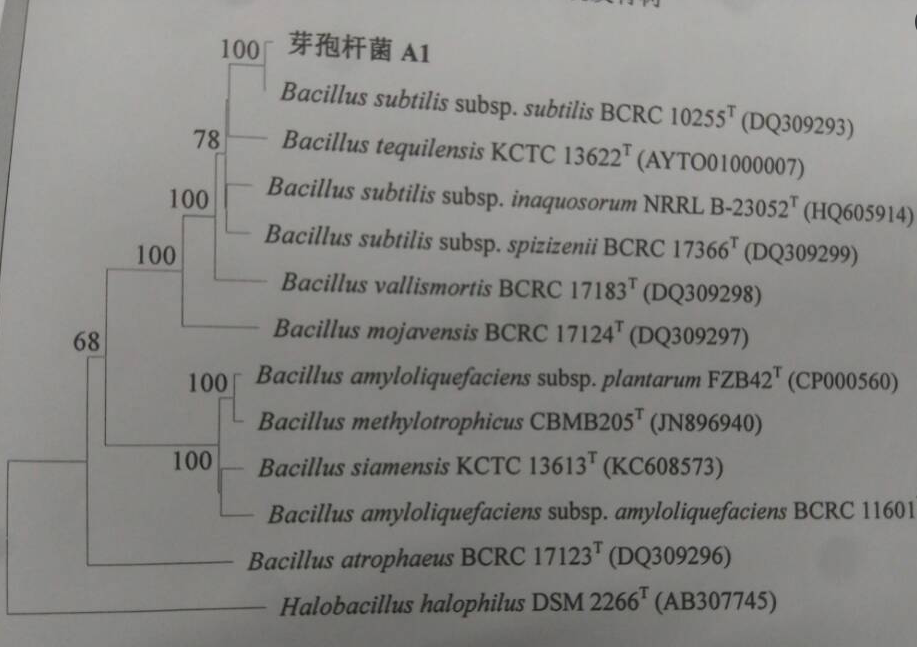


Morphological, biochemical tests and sequence analysis showed that the identified strain was ***Bacillus subtilis subsp.***
